# Lack of *SF3B1* R625 mutations in cutaneous melanoma

**DOI:** 10.1186/1746-1596-8-87

**Published:** 2013-05-21

**Authors:** Bastian Schilling, Nicola Bielefeld, Antje Sucker, Uwe Hillen, Lisa Zimmer, Dirk Schadendorf, Michael Zeschnigk, Klaus G Griewank

**Affiliations:** 1 Department of Dermatology, University Hospital, University Duisburg-Essen, Hufelandstrasse 55, Essen 45147, Germany; 2 Department of Human Genetics, University Hospital, University Duisburg-Essen, Hufelandstrasse 55, Essen 45147, Germany

**Keywords:** Melanoma, *SF3B1*, Cancer genetics, Dermatology

## Abstract

**Background:**

Melanoma is a deadly disease affecting people worldwide. Genetic studies have identified different melanoma subtypes characterized by specific recurrently mutated genes and led to the successful clinical introduction of targeted therapies. Hotspot mutations in *SF3B1* were recently reported in uveal melanoma. Our aim was to see if these mutations also occur in cutaneous melanoma.

**Findings:**

We analyzed a cohort of 85 cutaneous melanoma including 22 superficial spreading, 24 acral-lentiginous, 36 nodular, and 3 lentigo-maligna melanomas. Exon 14 of *SF3B1*, containing the site of recurrent mutations described in uveal melanoma, was sequenced in all samples. Additionally, *NRAS* exon 1 and 2 and *BRAF* exon 15 were sequenced in all, *KIT* exons 9, 11, 13, 17, and 18 in 30 samples. High numbers of *BRAF* and *NRAS* mutations were identified with frequencies varying according to melanoma subtype. None of the samples were found to harbor a *SF3B1* mutation.

**Conclusions:**

We conclude that recurrent mutations in codon 625 of *SF3B1* as reported in uveal melanoma are not present in most types of cutaneous melanoma. This highlights the genetic differences between cutaneous and uveal melanoma and the need for subtype specific therapeutic approaches.

## Introduction

Malignant melanoma is a devastating disease worldwide [1,2]. Curative management of melanoma is limited to the stage of localized disease. Once metastatic spread has occurred, prognosis of patients is poor. However, a number of promising new treatment regimens have been introduced recently, showing for the first time a therapy induced increase in overall survival [3,4].

Over the last couple of decades a number of genetic alterations have been identified in melanoma. Activating driver mutations in genes such as *NRAS*[5] and *BRAF*[6] were identified in cutaneous melanoma. Losses of tumor suppressors such as *CDKN2A* and *PTEN* have been well documented [7]. In uveal melanoma a different set of genes shows recurrent mutations, including *GNAQ* and *GNA11*[8,9], with activating mutations as well as in *BAP1*[10] showing inactivating mutations. The distinct mutation profiles of cutaneous and uveal melanoma are striking and support a model of different developmental pathways. However there is some overlap in tumor biology as ~80% of blue nevi, which are benign melanocytic tumors of the skin, also harbor *GNAQ* or *GNA11* mutations, [8] and *BAP1* mutations can be found in both cutaneous nevi and cutaneous melanoma [11-14].

Both genetic and immunohistological assays are becoming more and more relevant in determining the dignity and prognosis of melanocytic neoplasms [15-18]. Further refining which biomarkers are relevant in which settings should allow pathologists and clinicians to make more detailed diagnostic calls, leading to appropriate follow-up and treatment decisions.

Recently a recurrent mutation hotspot in *SF3B1* affecting codon 625 was found in 18.6% of uveal melanoma [19]. *SF3B1* mutations had been previously detected in myeloid malignancies such as CLL (chronic lymphoid leukemia) and MDS (myelodysplastic syndrome) [20,21] and also reported in breast cancer [22]. *SF3B1* is a splice factor, with mutations expected to result in altered pre mRNA splicing. However the exact target of altered splicing is unknown and might be cell type dependent [22].

The goal of our study was to analyze if *SF3B1* mutations not only play a role in uveal, but also in cutaneous melanoma.

## Material and methods

### Sample selection and histopathology

Cutaneous melanoma samples were obtained from the tumor bank of the Department of Dermatology, University Hospital, University Duisburg-Essen. The study was done with approval of the local ethics committee of the University of Duisburg-Essen.

### DNA isolation

10 μm-thick sections were cut from formalin-fixed, paraffin-embedded tumor tissues. The sections were deparaffinized and manually microdissected according to standard procedures. Genomic DNA was isolated using the QIAamp DNA Mini Kit (Qiagen, Hilden, Germany) according to the manufacturer’s instructions.

### Direct (Sanger) sequencing

Nested PCR was performed to amplify *BRAF* exon 15 and *NRAS* exon 1 and 2 and sequenced as previously described [23]. Sequencing of *KIT* exons 9, 11, 13, 17, and 18 was performed similarly. The first 120 base pairs of *SF3B1* exon 14 (covering codons 603–641) were sequenced using the forward primer – TGTTTACATTTTAGGCTGCTGGT and reverse primer – GCCAGGACTTCTTGCTTTTG. After purification with the QIAquick PCR Purification Kit (Qiagen) PCR products were used as templates for sequencing in both directions. The sequencing chromatogram files were examined, and mutations were identified using Chromas software (version 2.01, University of Sussex, Brighton, United Kingdom).

## Results

### Sample cohort

The cohort included tumors from 51 males and 34 females, including 22 superficial spreading, 24 acral-lentiginous, 36 nodular, and 3 lentigo-maligna melanomas, with an average Breslow tumor thickness of 3.62 mm. The average thickness between subtypes varied; acral-lentiginous melanoma (ALM) = 4.54 mm, nodular melanoma (NM) = 4.47 mm, superficial spreading melanoma (SSM) = 1.9 mm and lentigo maligna melanoma (LMM) = 0.53 mm.

### *NRAS*, *BRAF,* and *KIT* mutations

We analyzed 85 cutaneous melanomas in total. *BRAF* Exon 15 and *NRAS* Exon 1 and 2 were analyzed for presence of mutations by Sanger sequencing (Table 1). We identified 36 *BRAF* mutations (35 p.V600E, 1 p.V600K) and 19 *NRAS* mutations (11 p.Q61K, 3 p.Q61L, 5 p.Q61R). In total, 65% of tumors showed either a *BRAF* or *NRAS* mutation (42% *BRAF*, 22% *NRAS*). As reported previously, the mutations were found to be mutually exclusive. Prevalence of *BRAF* and *NRAS* mutations varied by histologic subtype; ALM - 33% *BRAF*, 13% *NRAS*, NM - 42% *BRAF*, 25% *NRAS*, and SSM - 60% *BRAF*, 23% *NRAS* mutations. Presence of *KIT* mutations was analyzed in 30 cases. 18 of these were in ALM in which the highest percentage of *KIT* mutations would be expected (18/24 ALM total = 75%) [24-26]. One ALM sample was found to harbor a p.N505H (c.1513A > C) mutation/variant. We further analyzed 7 NM, 4 SSM, and 1 LMM, not identifying any *KIT* mutations.

**Table 1:** Table of sequencing results

	**Oncogene mutation status**							**SF3B1**			
	**Total**	**WT**		**BRAF V600**		**NRAS Q61**		**NA**		**WT**	
	**Count**	**Count**	**%**	**Count**	**%**	**Count**	**%**	**Count**	**%**	**Count**	**%**
SSM	22	4	18.2%	13	59.1%	5	22.7%	1	4.5%	21	95.5%
NM	36	12	33.3%	15	41.7%	9	25.0%	3	8.3%	33	91.7%
ALM	24	13	54.2%	8	33.3%	3	12.5%	0	.0%	24	100.0%
LMM	3	1	33.3%	0	.0%	2	66.7%	0	.0%	3	100.0%

*WT*, Wildtype; *SSM*, Superficial spreading melanoma; *NM*, Nodular melanoma; *ALM*, Acral lentiginous melanoma; *LMM*, Lentigo maligna melanoma. *NA*, Not available (amplification failed or sequence reads were ambiguous).

### *SF3B1* analysis

**Figure 1:**
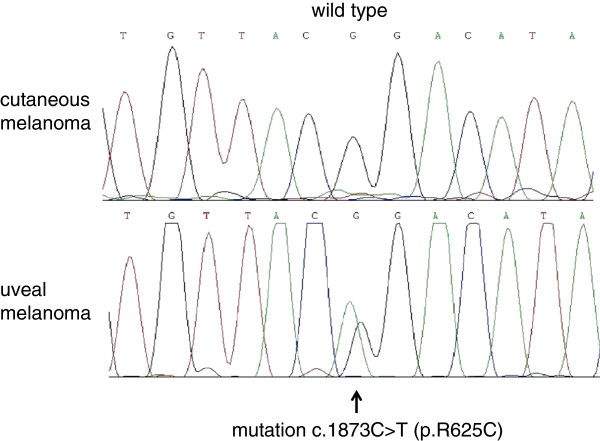
**Example of *SF3B1 *sequencing.** Shown are representative examples of *SF3B1* exon 14 sequencing, with a wild type sequence of a cutaneous melanoma on the top and a corresponding codon 625 mutation of a uveal melanoma on the bottom.

The first 120 base pairs of exon 14, containing the location of the known hotspot mutation at codon 625, were sequenced in all 85 samples. In four samples amplification failed or sequence reads were ambiguous. None of the remaining 81 samples showed a mutation in *SF3B1* as seen in a control sample from a uveal melanoma (Figure 1).

## Discussion

Genetic classification of different melanoma subtypes has become very important, especially with the introduction of effective therapies targeting genetic alterations such as *BRAF*[3,4] and *KIT* mutations [25]. A detailed understanding of the genetic events occurring in different tumors will most likely prove critical to further improving the therapeutic modalities for metastasized melanoma patients.

The distribution of activating oncogene mutations in *BRAF* and *NRAS* in our cohort is comparable to those reported elsewhere [7]. Overall 65% of melanoma had a *BRAF* or *NRAS* mutation in a mutually exclusive pattern. Of the three melanoma subtypes analyzed in considerable numbers (SSM, NM, ALM), percentages of *BRAF* and *NRAS* mutations combined were highest in SSM reaching 82%, lower in NM with 67% and lowest in ALM with 46%. The *KIT* mutation/variant identified in an ALM sample led to a p.N505V change. This is not reported to be a frequent mutation in cutaneous melanoma [27]. However p.N505H (c.1513A > C) is listed as a “variant of unknown origin” in a gastrointestinal stromal tumor in the COSMIC database [28]. The cutaneous ALM sample lacked mutations in *BRAF* or *NRAS* which could support a potential relevance, as typically *KIT* mutations are found to be mutually exclusive with *BRAF* and *NRAS* mutations [26]. The p.N505H (c.1513A > C) change could however also represent a rare germ-line variant, which we could not check as corresponding normal DNA was not available.

We obtained high quality sequencing results allowing analysis of exon 14 and in particular codon 625 of *SF3B1* in 81 samples and found no mutations. This argues against a major role for *SF3B1* in tumorigenesis or progression of cutaneous melanoma. In uveal melanomas, mutations were primarily found in tumors with a favorable prognosis [19]. Future studies could analyze if *SF3B1* mutations occur in benign cutaneous melanocytic tumors (nevi) or potentially in sites other than in codon 603–641 of exon 14 of *SF3B1*.

In recent years genetic analyses identified a number of key genes involved in melanoma formation or progression. Interestingly, almost all of those described in cutaneous melanoma are not known to be relevant in uveal melanoma [29,30]. In contrast, genetic alterations in uveal melanoma such as *GNAQ* and *GNA11* mutations were also found in selected cases of cutaneous melanoma and are frequently found in blue nevi (benign cutaneous melanocytic tumors) [9]. *BAP1* inactivating mutations are found in cutaneous nevi and melanoma, although considerably less frequently than in uveal melanoma [12]. Our current study would signify that *SF3B1* mutations, occurring in almost 20% of uveal melanoma, [19] do not play a major role in cutaneous melanoma. We believe this highlights once more the genetic differences between uveal and cutaneous melanoma and the need for development of melanoma subtype specific therapies.

## Abbreviations

ALM: Acral-lentiginous melanoma; SSM: Superficial spreading melanoma; NM: Nodular melanoma; LMM: Lentigo maligna melanoma.

## Competing interests

Dirk Schadendorf is on the advisory board or has received honararia from Roche, Genetech, Novartis, Amgen, GSK, BMS, Boehringer Ingelheim, and Merck. All other authors have nothing to declare.

## Authors’ contributions

Literature search: BS, LZ, MZ, DS, KGG Study design: BS, AS, DS, MZ, KGG Data collection: NB, AS, BS, UH, KGG Data analysis: BS, NB, AS, KGG Data interpretation: BS, LZ, DS, MZ, UH KGG Manuscript writing: all authors. All authors read and approved the final manuscript.
